# Editorial: Insights in public mental health: 2022

**DOI:** 10.3389/fpubh.2024.1360259

**Published:** 2024-02-08

**Authors:** Wulf Rössler

**Affiliations:** Department of Psychiatry and Psychotherapy, Charité University Medicine Berlin, Berlin, Germany

**Keywords:** public mental health, epidemiology, services research, intervention, stigma, traditional healing

In the continuation of our previous successful Research Topic, “*Insights in Public Mental Health*,” we now delve into the developments of the year 2022. Public Mental Health research has achieved notable progress in recent years. Our current focus revolves around novel perspectives and promising prospects within the Public Mental Health field. This Research Topic of articles aims to inform and inspire researchers in this domain.

At the forefront of Public Mental Health research lies the fundamental question of the ultimate goal of public healthcare. In a perspective article by Jackson et al., the “wellbeing epidemic” takes center stage. The authors explore the controversial concept of wellbeing and its implications for future health policies. Considering the historical context and the influence of Neoliberalism, they present a research agenda to scrutinize this concept.

Traditionally, the inquiry into individuals developing mental problems has mainly focused on vulnerability. However, recent Public Mental Health research has introduced another perspective: resilience. In their study, Lui and Duan demonstrate that individuals perceiving themselves as self-efficient, particularly against the backdrop of perceived social resources, play a crucial role in resilience. The study emphasizes the importance of instilling hope, a long-standing element in psychiatric rehabilitation.

Diagnostic issues remain a key interest in Public Mental Health, influencing how we conceptualize mental disorders and structure our healthcare systems. Mewes addresses the recent developments in psychological factors related to medically unexplained symptoms. These symptoms, often evolving into chronic conditions under the diagnosis of somatoform disorders, present challenges in treatment, leading to high healthcare costs.

The foundation of any treatment lies in a diagnosis, followed by the selection of the most appropriate treatment approach. However, not all patients within the same diagnostic class respond equally to treatments, and non-diagnostic factors further influence therapeutic settings. Thege et al. argue for more systematic research on trans-diagnostic factors influencing psychotherapeutic treatment outcomes, promoting a transition toward more patient-centered care.

Exploring the intersection of Occupational Psychology and Public Mental Health, issues related to shift work emerge as a significant concern. Heywon et al. identify pathways from depression, sleep, and cognition to work performance in shift and non-shift workers. Given that 10% to 40% of the working population worldwide is involved in shift work, addressing mental health problems arising from this practice is imperative.

The interface between society and Public Mental Health brings attention to “Intimate Partner Violence,” associated with multiple adverse health outcomes. Ortega-Ceballos et al. assess the association between Intimate Partner Violence and psychological distress mediated by substance use in a representative population sample. Their findings contribute valuable insights for designing public health policies addressing the prevention of Intimate Partner Violence and its mental health consequences.

For years, Public Mental Health research focused on evaluating service organization and structures within national contexts. However, generalizability to an international context became a priority. This article collection showcases interventions applicable in international environments.

Hill et al. describe an outreach intervention approach for those bereaved by suicide, emphasizing practical support in addition to psychological interventions. Fabel et al. investigate the implementation of Soteria-elements in psychiatric hospital acute wards, demonstrating positive outcomes. de Smet et al. analyze falls in psychiatric hospitals, particularly affecting older adult patients.

Standardized measures are crucial for international comparisons in Public Mental Health. Austria-Corrales et al. evaluates the validity and psychometric properties of a Spanish-language online version of the Columbia-Suicide Severity Rating Scale in a Mexican sample. This validation contributes to introducing the instrument in Latin American countries.

Psychiatric epidemiology plays a vital role in Public Mental Health research. Two publications from Germany address mental health and COVID-19-related epidemiological questions. Mauz et al. analyze representative data demonstrating the detrimental impact of COVID-19 on the mental health of the general population. Baldovski et al. assess attitudes and vaccination intentions among German University students and the general population.

Finally, to address the underrepresentation of the Global South in published Public Mental Health research, a narrative review by Ogunwale et al. examines the cultural framework for indigenous mental healthcare in Nigeria. The review discusses stigmatization and human rights abuses within the context of public mental health.

This Research Topic comprises 13 articles that exemplify significant contributions to Public Mental Health research. These articles not only benefit their respective healthcare settings but also provide insights applicable to a broader context. They are theoretically intriguing and uphold a high methodological standard, guiding the direction for future Public Mental Health research.

## Author contributions

WR: Writing – original draft.

